# A study of MD-PhD pre-health advising identifies challenges to building a robust MD-PhD applicant pool

**DOI:** 10.1172/jci.insight.185839

**Published:** 2025-04-08

**Authors:** Amara L. Plaza-Jennings, Christie B. Ryba, Jessica Tan, Jennifer E.L. Diaz, Grace E. Mosley, Talia H. Swartz, Margaret H. Baron, Robert Fallar, Valerie Parkas

**Affiliations:** 1 Medical Scientist Training Program, The Icahn School of Medicine at Mount Sinai, New York, New York, USA.; 2 Department of Hematology/Oncology, Washington University in St. Louis, St. Louis, Missouri, USA.; 3 Department of Medicine,; 4 Department of Hematology and Medical Oncology,; 5 Department of Medical Education, and; 6 Department of Internal Medicine, Division of Infectious Diseases, The Icahn School of Medicine at Mount Sinai, New York, New York, USA.

## Abstract

MD-PhD programs provide interdisciplinary training in medicine and research. Undergraduate pre-health advisors (PHAs) play a critical role in counseling prospective applicants, yet there have been no studies to our knowledge of MD-PhD pre-health advising. Here we surveyed 280 PHAs from US colleges and universities using both qualitative and quantitative measures that assessed their real-world advising behaviors as well as standardized evaluation of 1 of 2 fictional MD-PhD applicants, identical except for gender. We identified 3 factors that influenced advising behaviors: experience advising MD-PhD applicants, attitudes toward MD-PhD programs, and gender bias. Those PHAs with less experience and who held negative attitudes toward MD-PhD programs were less likely to initiate discussions about MD-PhD programs with qualified applicants and less likely to recommend the fictional applicants apply to MD-PhD programs. Finally, there was subtle gender bias that favored the male applicant. PHAs face challenges in advising MD-PhD applicants because there are relatively few MD-PHD applicants overall and there is a lack of resources to guide them. Addressing these challenges by strengthening collaborations with PHAs and providing comprehensive information about the value of and applicant qualifications for MD-PhD programs is crucial to enhancing MD-PhD advising, mitigating effects of bias, and expanding the pool of qualified applicants.

## Abstract 2

Pre-health advisors can impact the make-up of the MD-PhD applicant pool. We found that their experience and opinions of MD-PhD programs impact advising behaviors.

## Introduction

MD-PhD dual-degree programs provide interdisciplinary training in medicine and research to future physician-scientists, who are poised to perform clinically informed research and translate this research into advances in patient care (1–4). Pre-health advisors (PHAs) are an essential resource for students considering applying to MD or MD-PhD programs and can impact students’ decisions. This has been recognized in the context of MD applicants, where advising is especially critical for students from underrepresented backgrounds whose informal networks may not include individuals with knowledge about pre-medical pathways (5–8). In 2023–2024 there were only 1,795 MD-PhD applicants (3.4% of the total MD applicant pool) (9, 10). Therefore, it is not surprising that little is known about MD-PhD pre-health advising and that most PHAs are likely to have little experience with or knowledge of advising MD-PhD applicants (11, 12).

Studies are needed to understand the state of MD-PhD advising because, despite a desire to grow the physician-scientist workforce (13–15), applications to MD-PhD programs have remained relatively stagnant. Over the past decade, the number of applicants to MD-PhD programs has decreased by 7%, while the number of applicants to MD programs increased by 10% over the same period (9, 10, 16, 17). Studies of matriculated MD and MD-PhD students have identified gaps in pre-health advising that hinder prospective MD-PhD applicants (12, 18). Students voiced a desire for more information about MD-PhD programs and believed that PHAs could not provide sufficient information (12). Another study found that matriculated MD-PhD students who took gap years identified PHAs as the most influential outside source in deciding to take time off (18). Thus, there is evidence that PHAs may be less familiar with advising MD-PhD applicants and that they can impact applicants’ decisions. However, no studies to our knowledge have surveyed the PHAs to understand their knowledge level and factors that impact their advising of MD-PhD applicants.

Here we surveyed PHAs from across the country to understand how they evaluate and advise MD-PhD applicants. They were asked to evaluate 1 of 2 fictional MD-PhD applicants, identical except for gender, and answer questions about how they advise students in the real world. We identified 3 factors that significantly affected advising behaviors, level of experience with MD-PhD advising, negative attitudes toward MD-PhD programs, and gender bias. Our study is the first national PHA survey to our knowledge to investigate these issues and reveals opportunities for MD-PhD programs and national organizations to better support PHAs to achieve a robust MD-PhD applicant pool.

## Results

### Study participant characteristics.

Initially, 1,918 PHAs were sent a survey invitation and asked to evaluate the application materials of a fictional undergraduate student who has ostensibly shown interest in applying to MD-PhD programs. The PHAs were randomly assigned to receive identical application materials except for name and associated gender pronouns (Samuel, he/him/his or Samantha, she/her/hers). A total of 280 PHAs completed the survey, with 140 (50%) reviewing the male student and 140 (50%) reviewing the female student. Respondent demographics were not significantly different across the categories measured between the 2 groups (Table 1). Both groups had a diverse breakdown of home institution type, location, and size.

Relative to the pool of PHAs invited to complete the survey, the study participants came from institutions with a similar geographic distribution. The geographic breakdown for all PHAs invited to participate in the survey was 13% West, 32% Midwest, 8% Southwest, 23% Southeast, and 24% Northeast. Survey participants came from institutions that were 16% in the West, 26% Midwest, 7% Southwest, 26% Southeast, and 25% Northeast. Similarly, 15% (*n* = 42) of respondents came from institutions with MD-PhD programs, which matches the overall proportion of PHAs from institutions with MD-PhD programs who were invited to participate (14%).

### Overall applicant recommendations.

**Figure 1:**
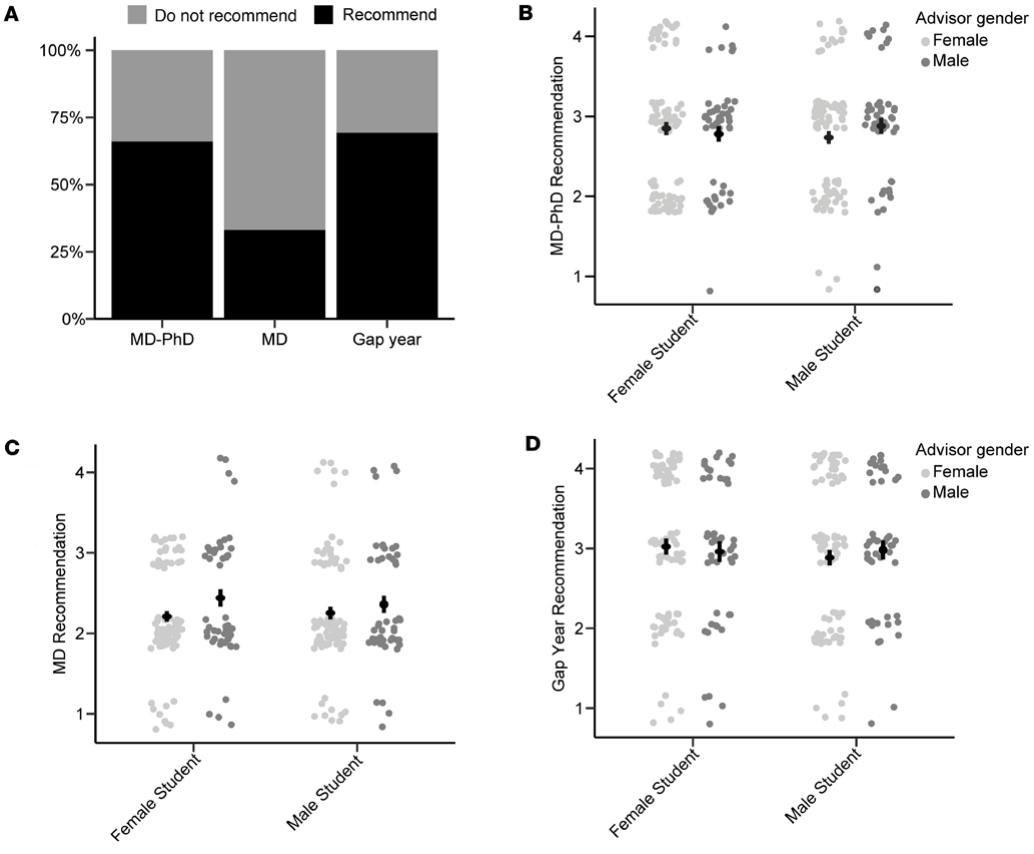
Overall advising recommendations of fictional applicants. (**A**) Graph showing the percentages of PHAs who recommended or did not recommend the fictional applicants apply to the indicated programs. (**B**–**D**) Dot plots with average and standard error of program recommendation score grouped by the gender of the PHA and of the applicant. Data points have been jittered along the *x*- and *y*-axes for better visualization. (**B**) MD-PhD recommendation score. *P* = 0.17. (**C**) MD recommendation score. *P* = 0.06. (**D**) Gap year recommendation score. *P* = 0.88. Differences were assessed using 2-way ANOVA.

We first looked at the overall recommendations that the PHAs made for the fictional applicants. We found that they recommended the applicants apply to MD programs 33% of the time, MD-PhD programs 66% of the time, and to take a gap year(s) prior to applying to MD-PhD programs 69% of the time (Figure 1A). We then looked to see whether there was any effect of PHA or applicant gender on MD-PhD recommendation. A 2 (student gender: male, female) by 2 (advisor gender: male, female) between-applicant ANOVA showed no significant effect of either applicant gender or PHA gender on the likelihood of recommending the applicant apply to MD-PhD programs (MD-PhD recommendation score, Figure 1B). Similarly, we saw no effect of PHA or applicant gender on MD or gap year recommendation scores (Figure 1, C and D).

### Gaps in MD-PhD advising experience and knowledge.

We hypothesized that level of familiarity and experience with pre-health advising for MD-PhD applicants may impact advising. Of the study respondents, 15% (*n* = 43) of PHAs reported having significant experience advising MD-PhD applicants, 44% (*n* = 123) reported moderate experience, 38% (*n* = 105) minimal experience, and 3% (*n* = 9) no experience (Table 1). Furthermore, 14% of unprompted, qualitative responses identified a desire for more guidance on advising MD-PhD applicants and indicated uncertainty about how to judge whether an applicant is qualified (*n* = 10 out of 69; Table 2, “Uncertainty surrounding MD-PhD advising”). Eight of these 10 responses came from individuals with minimal or moderate experience advising MD-PhD applicants.

Lack of familiarity with MD-PhD admissions was also reflected in qualitative responses related to criteria for successful MD-PhD applicants (Table 2, “Criteria for successful application”). First, there was a lack of consistency between respondents as to what the most important qualifications were. Research experience was identified as a key metric, but there was still a high degree of variability regarding what individuals believed constituted sufficient experience. Many PHAs referenced a specific need for publications, which a survey of program directors has shown is not essential for admission (18). There was also a lack of consensus on how much clinical and volunteer experience is needed.

**Figure 2:**
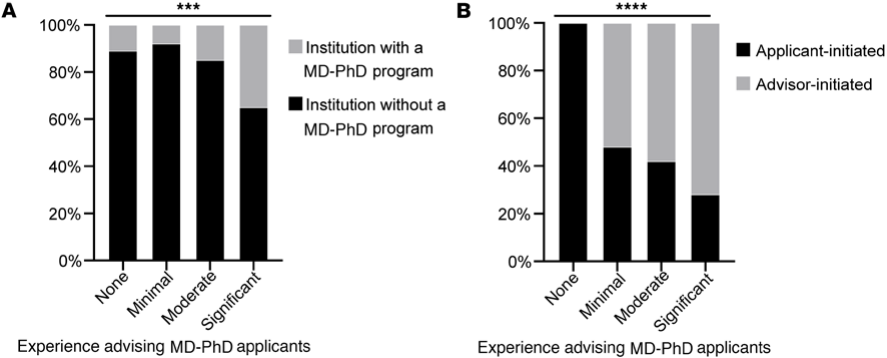
Experience advising MD-PhD applicants. (**A**) The percentages of respondents at institutions with or without MD-PhD programs, grouped by experience advising MD-PhD applicants. (**B**) The percentages of respondents who discuss MD-PhD programs when applicants express interest (applicant-initiated) and when an applicant is qualified but has not expressed interest (advisor-initiated), grouped by experience advising MD-PhD applicants. ***P < 0.001, ****P < 0.001 by χ^2^ test.

We next considered whether the presence of an MD-PhD program at the PHA’s home institution could impact the level of experience advising MD-PhD applicants. We found that PHAs with significant experience advising MD-PhD applicants were more likely to work at institutions with an MD-PhD program (Figure 2A). We also found that level of experience impacted real-world advising behaviors. PHAs were asked in what contexts they discuss MD-PhD programs with applicants. Almost all (95%, *n* = 266) PHAs reported discussing MD-PhD programs with applicants when the applicant expressed interest (“applicant-initiated” discussion). However, only half (55%, *n* = 153) of PHAs reported discussing MD-PhD programs with qualified applicants who have not explicitly expressed interest (“advisor-initiated” discussion). PHAs with less experience advising MD-PhD applicants were significantly less likely to proactively discuss MD-PhD programs with qualified applicants (Figure 2B). These results demonstrate a gap in PHA knowledge and experience that impacts their advising of potentially qualified MD-PhD applicants.

### Attitudes toward MD-PhD programs impact advising.

Beliefs that MD-PhD programs are not worth the time investment or are not effective for training physician-scientists are common (12, 19, 20). We wanted to quantify the prevalence of these beliefs among PHAs and understand whether they impact advising. To test for potential negative attitudes toward MD-PhD programs, we asked respondents to rate their agreement with the statement “I think MD-PhD programs have more advantages than disadvantages.” Most PHAs (77%, *n* = 216) agreed or strongly agreed (designated as having positive attitudes toward MD-PhD programs). However, a substantial proportion (23%, *n* = 64) of PHAs believed that MD-PhD programs have more disadvantages than advantages (strongly disagree or disagree, designated as having negative attitudes toward MD-PhD programs). These beliefs were reflected in the qualitative survey responses, where several respondents reported discouragement of prospective MD-PhD applicants they had advised based on their experiences and attitudes toward MD-PhD programs (Table 2, “Overt discouragement”). Specifically, we saw that some PHAs believe that it is better to acquire either an MD or PhD and seem to suggest that the combined degree leads to an inferior version of both degrees.

**Figure 3:**
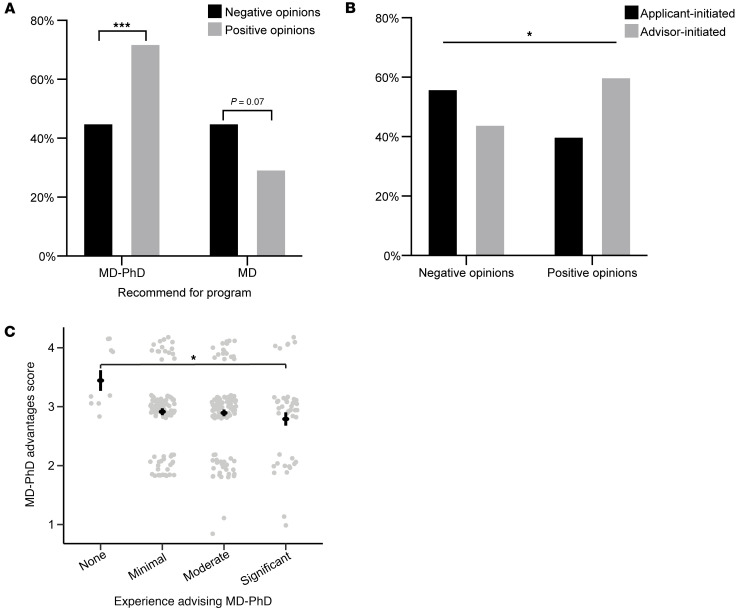
Attitudes toward MD-PhD programs impact advising. (**A**) Percentage of respondents who recommended (agree or strongly agree) the applicant apply MD-PhD or MD programs, grouped by their attitudes toward MD-PhD programs. Respondents who agreed or strongly agreed with the statement “I think MD-PhD programs have more advantages than disadvantages” were grouped as positive attitudes toward MD-PhD programs. Respondents who disagreed or strongly disagreed were grouped as negative attitudes toward MD-PhD programs. Welch’s *t* test (MD-PhD: *P* = 0.0001, MD: *P* = 0.07). (**B**) The percentages of respondents who discuss MD-PhD programs when applicants express interest (applicant-initiated) and when an applicant is qualified but has not expressed interest (advisor-initiated), grouped by attitudes toward MD-PhD programs as defined in **A**. Fisher’s exact test (*P* = 0.02). (**C**) Dot plot with average and standard error of MD-PhD program advantages score grouped by level of experience advising MD-PhD applicants. Data points have been jittered along the *x*- and *y*-axes for better visualization. Overall ANOVA *P* = 0.049; post hoc Tukey’s test is shown for the only significant comparison (*P* = 0.027). *P < 0.05, ***P < 0.001.

Furthermore, the PHAs’ attitudes about MD-PhD programs significantly affected how they advised the fictional applicants and their real-world behaviors. PHAs with negative attitudes toward MD-PhD programs were less likely than PHAs with positive attitudes to recommend the fictitious applicant apply to MD-PhD programs (Figure 3A). There was also a trend that PHAs with negative attitudes toward MD-PhD programs more often recommended that the fictional applicant apply to MD-only programs. Similarly, a significantly lower proportion of PHAs with negative attitudes toward MD-PhD programs reported initiating discussions about MD-PhD programs with qualified applicants than those with positive attitudes (Figure 3B). The only PHA demographic that had a significant association with MD-PhD program attitudes was experience advising MD-PhD applicants (Figure 3C). We found that PHAs with significant experience were more likely to hold negative attitudes toward MD-PhD programs than those with no experience. These data demonstrate that negative attitudes towards MD-PhD programs are prevalent among PHAs and impact how they advise applicants.

### Gender bias impacts applicant evaluation.

**Figure 4:**
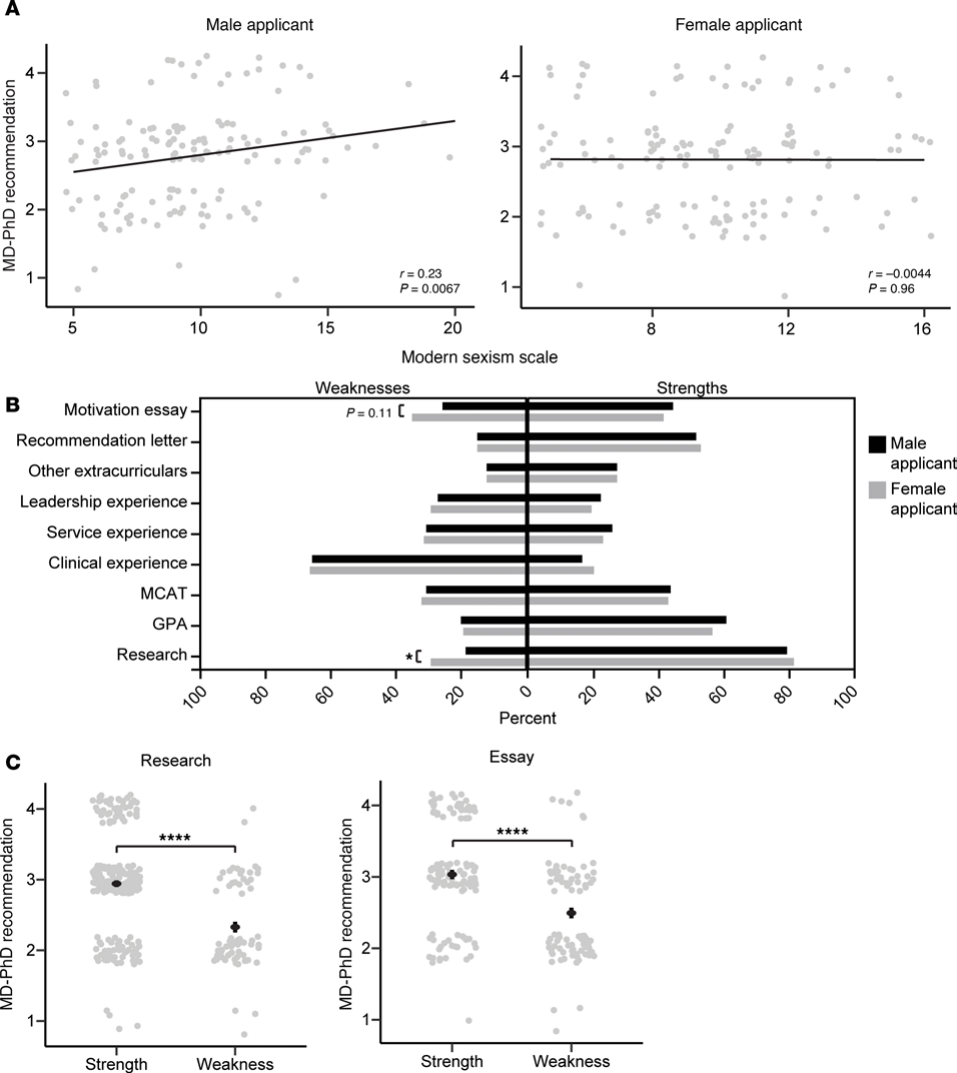
Gender bias impacts applicant evaluation. (**A**) Scatterplots of the MD-PhD recommendation score as a function of the total score on the Modern Sexism Scale, with the linear regression line for the male and the female applicants. Spearman’s correlation: statistics shown in figure. (**B**) The percentage of PHAs who checked each application category as a strength or weakness grouped by whether they evaluated the male or the female applicant. Research as a weakness: Student’s *t* test (research as a weakness, *P* = 0.05; motivation essay as a weakness, *P* = 0.11). (**C**) Dot plot with average and standard error of MD-PhD recommendation score grouped by PHAs who checked research or essay as a strength or a weakness of the applicant. Welch’s *t* test (research, *P* < 0.0001; essay, *P* < 0.0001). Data points have been jittered along the *x*- and *y*-axes for better visualization. *P < 0.05, ****P < 0.0001.

Given that the absence of clear selection criteria can increase the impact of bias in decision making, we wanted to investigate how unintentional PHA bias might impact advising behavior (21). We assessed whether or not there was an effect of applicant or PHA gender on MD-PhD pre-health advising. Since there was no overall effect of applicant or PHA gender (Figure 1B), we next assessed whether gender bias, as measured by the abbreviated Modern Sexism Scale, had an impact on advising. Overall, male PHAs averaged significantly higher scores (mean = 10.22) than female PHAs (mean = 9.445), indicating greater gender bias (*P* = 0.04, Welch’s *t* test). Furthermore, a higher Modern Sexism Scale score was correlated with higher MD-PhD recommendation scores for the male student (*P* < 0.001), while there was no significant correlation for the female applicant (Figure 4A).

We also observed gender differences in what PHAs identified as strengths or weaknesses of the applicants’ qualifications. Research experience was significantly more likely to be considered a weakness of the female student’s application than the male student’s (*P* = 0.05, Figure 4B). In accordance with this, 19% (*n* = 15 out of 81) of qualitative feedback for the female applicant specifically referenced a lack of research publications as a barrier to admission, whereas only 10% (*n* = 7 out of 71) of the responses for the male applicant did so. Additionally, there was a trend that the MD-PhD motivation essay was considered a weakness more often for the female applicant than for the male applicant (*P* = 0.11, Figure 4B). Importantly, PHAs’ perceptions of the applicants’ research experience and essay as a strength or a weakness significantly affected their recommendation to apply to MD-PhD programs (*P* < 0.001 for both, Figure 4C). In all, these findings suggest that subtle gender biases can impact applicant evaluation and advising.

## Discussion

PHAs are resources and gatekeepers for prospective MD and MD-PhD applicants. However, given that the MD-PhD applicant pool is only 3.4% of the total MD pool, most PHAs are likely to advise relatively few applicants applying to MD-PhD programs (9, 10). There are important differences in advising MD-PhD and MD applicants, such as the necessary qualifications and ultimate career goals. Still, there have yet to be studies characterizing MD-PhD pre-health advising. Understanding MD-PhD pre-health advising is important, given the desire to increase the size and diversity of the MD-PhD workforce (1, 2, 13–15, 22). In this study, we identified 3 factors that affected advising behaviors, PHA experience, attitudes toward MD-PhD programs, and gender bias, underscoring specific opportunities to better support and partner with PHAs.

Overall, PHAs were most likely to recommend that the fictional applicant take time off prior to applying to MD-PhD programs. This is interesting in light of recent work showing an increase in the number of students taking gap years prior to beginning MD-PhD training, thereby lengthening the overall time prior to finishing training (18). Our findings show that PHAs may be contributing to this trend, when not all students require gap years. Furthermore, we observed that most PHAs have minimal to moderate experience advising these applicants. However, more concerningly, we found that they also felt they lacked information on how to advise these applicants, a more prevalent feeling among PHAs with less experience. Our results suggest that this lack of experience and knowledge negatively impacts the MD-PhD applicant pool by preventing PHAs from discussing MD-PhD programs with qualified applicants who may not be aware these programs exist. Institutions without MD-PhD programs are especially affected, as their PHAs report having less experience advising MD-PhDs. While this finding is unsurprising, it is still concerning because we recognize that a diverse group of MD-PhD trainees must come from diverse undergraduate institutions. A study that performed in-depth interviews with PHAs found that they face numerous challenges in advising students, including a lack of formal training, high student-to-PHA ratios, and having multiple responsibilities beyond advising (23). The workload and student/PHA ratios were higher at larger, less well-resourced public universities. Additionally, not all colleges or universities have dedicated PHAs. This fits with our results and points to structural challenges at the college or university level that impact advising.

Negative opinions concerning the merits of MD-PhD programs are pervasive among individuals in biomedical fields and have been suggested to deter potential applicants (12, 19, 20). Here, we saw that a substantial proportion of PHAs had negative attitudes toward MD-PhD programs. When PHAs held these beliefs, they were less likely to recommend the applicants to MD-PhD programs and were less likely to initiate discussions about MD-PhD programs with qualified applicants. PHAs with negative opinions of MD-PhD programs may therefore contribute to the stagnation in number of MD-PhD applicants by discouraging applicants from MD-PhD programs. PHAs with significant experience were more likely to hold these negative beliefs than those with no experience. However, only 6 respondents had no experience advising MD-PhD applicants, so it is difficult to say how meaningful this result is. It is not clear what is driving this trend, but it may be that those PHAs with significant experience have had more negative outcomes in helping students apply to these programs, as some of the qualitative responses mentioned (Supplemental Table 1). MD-PhD programs are less well known than MD programs, and PHAs can help raise awareness among applicants for whom MD-PhD programs may be a good fit. Without external guidance, applicants by default self-select early on during college (Table 2, “Applicant self-selection”), potentially excluding qualified applicants with fewer connections to individuals in the biomedical fields.

Lack of experience evaluating MD-PhD applicants or uncertainty about applicant qualifications may lead to increased unintentional bias from PHAs. This has been seen in previous studies, where, in the absence of predefined selection criteria, evaluators shift their own selection criteria to align with their biases (21, 24). Gender bias was evident in evaluation of the students’ research experience, which is one of the most important aspects of the MD-PhD application and was an independent predictor of MD-PhD recommendation strength in the survey. Of note, respondents stated that a lack of research publications was a barrier to acceptance at twice the rate for the female applicant as they did for the male candidate. This is troubling for 2 reasons: (a) female applicants are disproportionately disadvantaged by this belief and (b) not all MD-PhD programs require publications for admission (25). Additionally, we observed an effect of sexism that favors the male applicant. These results fit in with a larger body of work showing that women are considered less qualified for positions in the biological sciences, even with the same qualifications as male candidates (26). We also saw a trend that the MD-PhD motivation essay was more often identified as a weakness of the female candidate. Both the essay and research experience are categories of the application without clear, objective metrics for success and which PHAs might have less experience evaluating, as they are specific to MD-PhD applicants. These types of ambiguous criteria have been shown to be prone to bias in college admissions (27). In contrast, quantitative metrics (MCAT score, GPA), although also potentially prone to biases (27), and typical MD qualifications (clinical experience) were rated similarly for both applicants. Though not explicitly tested here, it is likely that racial biases could have a similar impact for students from groups underrepresented in medicine. Notably, PHAs themselves have identified facing more challenges in advising students underrepresented in medicine (23), which merits further study.

One limitation of the study is that respondents were given limited information to judge the applicant that may only reflect part of the individual. We recognize that MD-PhD programs are only a good fit for some applicants, and determining which program is best for an applicant is a part of a nuanced advising process. Several qualitative responses voiced this concern, noting that advising is normally a holistic process involving extensive conversations (Supplemental Table 1). Secondly, the response rate for the survey was 15%, which may be considered low. However, this is a reasonable response rate given that this was an entirely voluntary survey with no incentives. Additionally, we saw that both the geographic distribution and proportion of individuals at institutions with MD-PhD programs were similar between respondents and all PHAs who received the survey, indicating that our respondent sample was representative. Finally, this study only examines one aspect of the physician-scientist pipeline. Still, efforts are needed throughout this process, including during and after MD-PhD training, to increase the size and diversity of the physician-scientist workforce.

Our study has identified several challenges facing PHAs that potentially impede the development of a robust MD-PhD applicant pool. Based on the challenges we identified, we have the following recommendations to enhance partnerships with PHAs: (a) We recommend expanding resources available on the American Association of Medical Colleges (AAMC) website, which several respondents identified as helpful (Supplemental Table 1). These resources should be designated for PHAs, which they are not currently, and include information about the MD-PhD career path, its value, and the qualifications of a successful applicant. Resources related to applicant qualifications should have clear guidelines focusing on research experience. (b) We recommend improved dissemination of resources and knowledge to the PHA community by the AAMC in partnership with the National Association of Advisors for the Health Professions (NAAHP). These efforts are already underway by the AAMC Great MD-PhD Section Communications Committee, which has been giving talks at national and regional NAAHP meetings; however, they can be expanded to increase their reach by partnering with the NAAHP to reach out directly to PHAs. (c) We propose that individual MD-PhD programs work to strengthen their relationships with individual PHAs, especially those from institutions without MD-PhD programs. Each MD-PhD program may have multiple “sister schools” with whom they maintain relationships. Studies of Historically Black Colleges and Universities have found higher rates of admittance to medical school when the PHAs had stronger ties to admissions officers, underscoring the importance of these relationships (6, 28). (d) Given the evidence that subtle gender bias impacts advising, we propose that colleges and universities should implement implicit bias training to mitigate these effects. The impact of implicit bias has long been recognized in medicine and implicit bias training has been shown to be effective in increasing self-identification of bias and bias reduction efforts (29, 30). (e) Finally, to reach more PHAs we also suggest the publication of articles and resources in PHA journals. Further studies, including interviews, with PHAs may help to better understand MD-PhD pre-health advising and how PHAs decide to guide students toward MD-PhD, research-focused MD, or PhD programs. Together, these efforts could help expand and improve the diversity of the MD-PhD applicant pool.

## Methods

### Survey development.

The survey was developed in-house based on a review of the literature and the authors’ own experiences. The survey consisted of 4 sections, after which it was impossible to navigate backward in the survey. These sections were: (a) their experiences and attitudes related to MD-PhD advising, (b) evaluation of a fictional MD-PhD applicant, (c) their views on gender equity using an abbreviated version of the Modern Sexism Scale, and (d) respondent demographic information (see complete survey in Supplemental Figure 1). Respondents were able to provide qualitative feedback in section 2, related to applicant feedback, and general feedback at the end of the survey.

For section 2 (applicant evaluation), respondents were randomly provided with 1 of 2 versions of the application material. The 2 versions were identical except for the name and corresponding gender pronouns of the applicant (Samuel, he/him/his; Samantha, she/her/hers). Based on the model of Moss-Racusin et al. (26), participants were told that the application materials came from a real student who was considering applying to MD-PhD programs and had provided their materials in exchange for the survey feedback. This was done to ensure that the PHAs believed there were consequences to their responses and gave the applicant adequate consideration. The application materials were developed in consultation with the Senior Associate Dean of Admissions at The Icahn School of Medicine at Mount Sinai and the Director of the MD-PhD Program at The Icahn School of Medicine at Mount Sinai to reflect a student with good, but not outstanding, qualifications as an MD-PhD applicant since it is more likely that respondent-specific factors might sway recommendations for applicants with ambiguous qualifications (26, 31, 32). The PHAs were asked to evaluate the students based on the provided application materials, which included a resume, an excerpt from a letter of recommendation from the student’s undergraduate thesis advisor, and an excerpt from the MD-PhD American Medical College Application Service essay answering the prompt “Please state your reasons for wanting to pursue the combined MD-PhD degree” (provided application materials in Supplemental Figures 2 and 3).

In order investigate how unintentional bias might impact advising behavior, we included an abbreviated version of the Modern Sexism Scale (33). This scale is a validated instrument to measure subtle forms of sexism. It was abbreviated by excluding 3 questions determined to be less relevant in the modern era (Supplemental Figure 1).

### Study participants.

The survey was emailed to 1,918 PHAs from 754 US colleges and universities between November 2018 and June 2019. A comprehensive list of every college and university by state was generated based on a database of PHA contact information from the Senior Associate Dean of Admissions at The Icahn School of Medicine at Mount Sinai, supplemented by a manual search (34). The manual search included anyone identified as a PHA on the institution’s website, any faculty identified as a member of a pre-health advising committee, and any pre-health advising office email accounts. At the time of survey closing, there were 280 completed surveys.

### Procedure.

All 1,918 PHA emails identified were randomized to receive the application materials of either the male or female fictional applicant. Study data were collected and managed using REDCap electronic data capture tools hosted at The Icahn School of Medicine at Mount Sinai (35, 36). Survey results were anonymous and contained no identifying information. They were downloaded from REDCap as an Excel file.

### Statistics.

In questions with a modified Likert scale, responses were converted to a numeric scale for analysis (strongly agree = 4, agree = 3, disagree = 2, strongly disagree = 1). As indicated in the figure legends, statistical tests were performed using an unpaired, 2-tailed *t* test, Mann-Whitney test, 1-way or 2-way ANOVA with Tukey’s multiple-comparison test, or Kruskal-Wallis with Dunn’s multiple-comparison test for post hoc analysis (GraphPad Prism 8). Spearman’s correlation was performed using RStudio 1.1.456 (https://posit.co/download/rstudio-desktop/). A *P* value of less than 0.05 was considered significant.

For the analysis of the qualitative responses, major themes were identified through content analysis of all responses. Two independent evaluators scored the qualitative responses by assigning them to one or more identified themes (see Supplemental Table 1 for a list of themes and complete scored qualitative responses). The final scoring of themes was identified by concordant evaluator responses. For discordant responses, evaluators reviewed and came to a consensus.

### Study approval.

This study was approved by the Institutional Review Board of The Icahn School of Medicine at Mount Sinai and deemed to be exempt as non–human participant research. All survey responses were anonymous and did not include any identifying information.

### Data availability.

Qualitative response data are available in Supplemental Table 1. Values for all figures are available in the Supporting Data Values file.

## Author contributions

ALPJ and CBR developed the survey, implemented the survey, analyzed the data, and wrote the manuscript. The order of the co–first authors ALPJ and CBR was decided based on alphabetical order. JT, JELD, and GEM assisted in the conceptualization of the study, development of the survey, and editing of the manuscript. THS, MHB, RF, and VP assisted in the development of the survey and editing of the manuscript. All authors read and approved the final manuscript.

## Supplementary Material

Supplemental data

Supplemental table 1

Supporting data values

## Figures and Tables

**Table 1:**
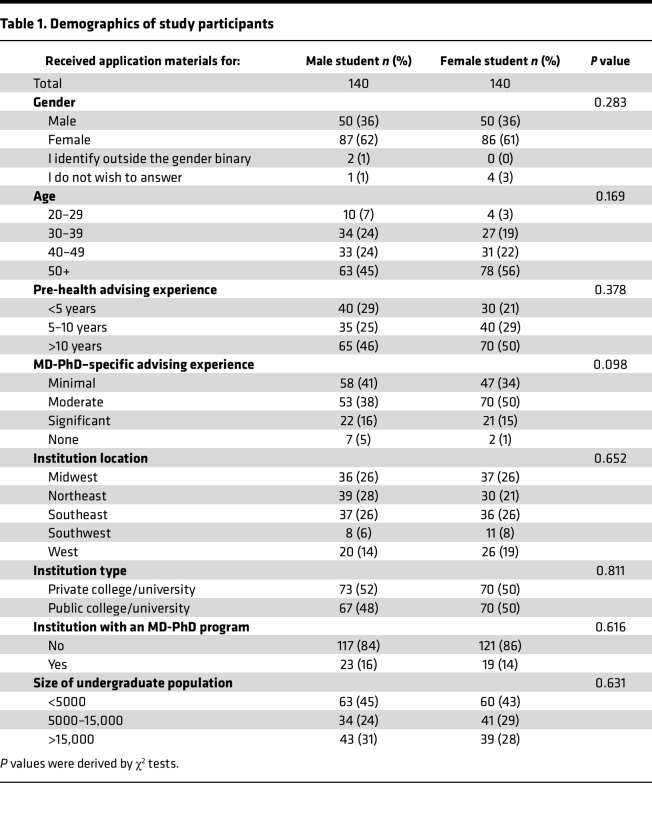
Demographics of study participants

**Table 2:**
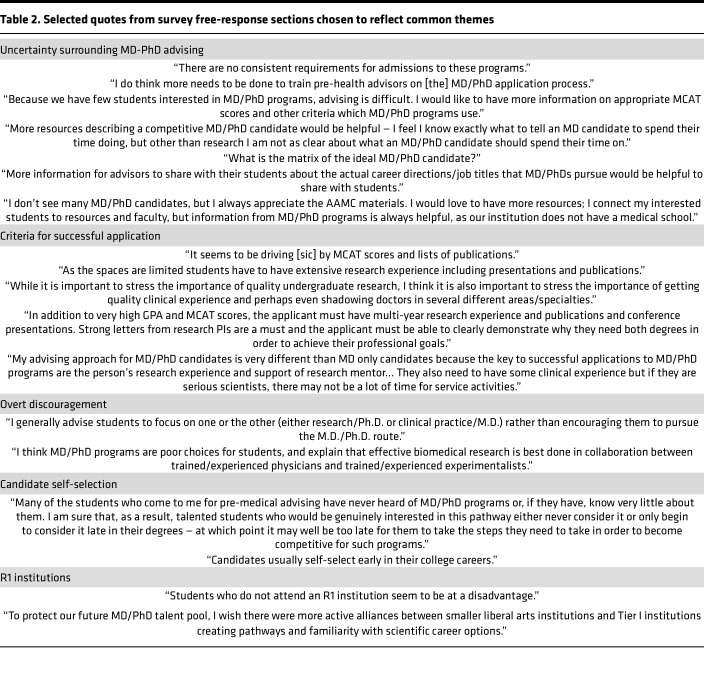
Selected quotes from survey free-response sections chosen to reflect common themes

## Version 1 04/08/2025

Electronic publication
