# Supplementary material for: A study of MD-PhD pre-health advising identifies challenges to building a robust MD-PhD applicant pool
Source: JCI Insight. 2025 Apr 8;10(7):e185839. doi: 10.1172/jci.insight.185839 (PMC11981617; doi:10.1172/jci.insight.185839)
Supplement: Supplemental data [file jciinsight-10-185839-s232.pdf]

**Supplemental Figure 1.** Complete survey

# MD/PhD Pre-Health Advising

Please complete the survey below. Thank you for your participation.

---

We are interested in understanding the factors that pre-health advisors consider the most important in MD/PhD applications and how pre-health advising for MD/PhD applicants varies.

We have compiled application materials from real students who are interested in applying to MD/PhD programs and are seeking advice as to whether this is a good choice for them. These students have volunteered their information in exchange for the feedback received in this study.

You will be reviewing the materials for one randomly-selected student and evaluating the student as if this was a person at your own school. Please answer the survey honestly; all responses are completely anonymous. The survey should take no more than 15 minutes.

Thank you for your time.

---

Below is the link to download the application information, including CV, letter of recommendation excerpt, and "Why MD/PhD" AMCAS excerpt. Download and read this information before proceeding.

[Attachment: "Application08.pdf"]

---

In what context are MD/PhD programs discussed with your students (select all that apply)?

- ☐ When students express interest
- ☐ When a student is qualified but has not expressed interest
- ☐ Never
- ☐ Other

---

Please specify: __________________________

---

Please rate your agreement with the following statement.

| | Strongly disagree | Disagree | Agree | Strongly agree |
|---|---|---|---|---|
| I think MD/PhD programs have more advantages than disadvantages. | ○ | ○ | ○ | ○ |

**This student is deciding whether or not to apply to MD/PhD programs. Please respond to the the following prompts as if you were advising this student in your office. Use the application materials provided (resume, essay excerpt, letter of recommendation excerpt) to guide your answers.**

**Please rate your agreement with the following statements.**

| | Strongly disagree | Disagree | Agree | Strongly agree |
|---|---|---|---|---|
| I am likely to recommend to this student that she applies to MD/PhD programs. | ○ | ○ | ○ | ○ |
| I am likely to recommend to this student that she applies to MD-only programs. | ○ | ○ | ○ | ○ |
| If she takes time off to bolster her resume, I would be more likely to recommend that she applies to MD/PhD programs. | ○ | ○ | ○ | ○ |

What are her strengths as an MD/PhD applicant?

- ☐ Research experience
- ☐ GPA
- ☐ MCAT score
- ☐ Clinical experience
- ☐ Service experience
- ☐ Leadership experience
- ☐ Other extracurriculars
- ☐ Letter of recommendation excerpt
- ☐ Essay excerpt
- ☐ Other

(Please select all that apply.)

What are her weaknesses as an MD/PhD applicant?

- ☐ Research experience
- ☐ GPA
- ☐ MCAT score
- ☐ Clinical experience
- ☐ Service experience
- ☐ Leadership experience
- ☐ Other extracurriculars
- ☐ Letter of recommendation excerpt
- ☐ Essay excerpt
- ☐ Other

(Please select all that apply.)

Please provide any other comments about the applicant.

__________________________________

**The following questions do not relate to the application materials.**

**Please rate your agreement with the following statements.**

| | Strongly disagree | Disagree | Neither agree nor disagree | Agree | Strongly agree |
|---|---|---|---|---|---|
| Discrimination against women is no longer a problem in the United States. | ○ | ○ | ○ | ○ | ○ |
| Women often miss out on good jobs due to gender discrimination. | ○ | ○ | ○ | ○ | ○ |
| Society has reached the point where women and men have equal opportunities for achievement. | ○ | ○ | ○ | ○ | ○ |
| It is easy to understand why women's groups are still concerned about societal limitations of women's opportunities. | ○ | ○ | ○ | ○ | ○ |
| Over the past few years, the government and the news media have been showing more concern about the treatment of women than is warranted by women's actual experiences. | ○ | ○ | ○ | ○ | ○ |

Please answer the following demographic questions. Your answers are anonymous.

What gender do you identify with?

○ Male
○ Female
○ I identify outside the gender binary
○ I do not wish to answer

What is your age?

○ 20-29
○ 30-39
○ 40-49
○ 50+

How long have you been a pre-health adviser?

○ < 5 years
○ 5-10 years
○ >10 years

| | None | Minimal | Moderate | Significant |
|---|---|---|---|---|
| How much experience do you have advising MD/PhD applicants? | ○ | ○ | ○ | ○ |

Where is your institution located?

○ West
○ Midwest
○ Southwest
○ Southeast
○ Northeast

| | |
|---|---|
| Which of the following describes your institution? | ◯ Private college/university<br>◯ Public college/university |
| What is the size of your institution's undergraduate population? | ◯ < 5000<br>◯ 5000-15000<br>◯ >15000 |
| Does your institution have an MD/PhD program? | ◯ Yes<br>◯ No |
| Do you have any other comments about MD/PhD pre-health advising? | __________________________________ |

**Supplemental Figure 2.** Male applicant materials

# SAMUEL [redacted]

University of Maryland, 2100 La Plata Hall, 2121 Farm Drive, College Park, MD 20742

[redacted]@umd.edu


## EDUCATION

**The University of Maryland**, College Park, MD 5/2018

B.S. in Biochemistry, Honors College

GPA 3.72

MCAT 512

**Calvert High School**, Prince Frederick, MD 6/2014

Salutatorian

GPA 3.9

## RESEARCH EXPERIENCE

**Maryland-HHMI Undergraduate Research Fellow** 5/2017-5/2018

Awarded fellowship to conduct a year of research under the direct mentorship of a faculty member, including a stipend of $6000 and up to $1000 in research supplies. Performed biochemical and cell biology experiments to study how signal transduction pathways affect T cell activation in cancer. The goal is to leverage this understanding for novel cancer immunotherapies. This research culminated in my senior thesis.

**Poster presentation** 9/2017

Presented work on senior thesis at CRI-CIMT-EATI-AACR International Cancer Immunotherapy Conference: Translating Science into Survival.

**Summer Internship Program in Biomedical Research** 6/2016-8/2016

Awarded internship to conduct 8-weeks of research at the National Institutes of Health. Identified and characterized genetic mutations in lymphoma and multiple myeloma using genomic and molecular approaches.

**Maryland Student Researcher** 9/2014-12/2014

Worked in a lab researching neurodevelopment in Drosophila for 6 hours per week. Set up crosses and prepared slides for imaging in a genetic screen.

## ACTIVITIES

**America Counts**, Co-president 9/2015-5/2018

Volunteered as a tutor for fourth grade students in local public schools teaching math for 3 hours twice a week. Selected to be co-president during junior year.

**University of Maryland Symphony Orchestra,** first chair cellist 9/2014-5/2018

Played cello in the Symphony Orchestra for all four years of college. Toured and competed with the orchestra internationally. Served as first chair cellist during my senior year.

**Shadowing** 6/2015-8/2015

Shadowed an oncologist for 3 hours per week. Observed patient visits and rounded in the hospital. Shadowed a neurosurgeon for 4 hours per week. Observed surgeries and patient visits.

## AWARDS/HONORS

**Banneker/Key Scholarship** 8/2015-5/2018

Awarded merit scholarship offered to 150 incoming Maryland freshmen that covers full tuition, room and board, and includes admission to the Honors College.

**Dean's List**

Awarded for 6 out of 6 semesters completed for having a GPA of greater than 3.5.

**Excerpt from thesis advisor’s letter of recommendation:**
“Samuel began working in my lab a year ago during his junior year and has been an enthusiastic and motivated addition to my team. Although he joined the lab later in his undergraduate career, I have witnessed Alex’s impressive ability to quickly learn complex biological concepts and a broad repertoire of laboratory techniques. His quick mind, collaborative spirit, and rigorous work ethic all demonstrate that he has the potential to become a truly successful physician scientist.”

**Excerpt from why MD/PhD statement:**
“I want to pursue a combined MD/PhD degree because having expertise in both the medical and research fields provides the best opportunity for advancement of the medical field. I shadowed an oncologist who also had a basic research laboratory. I saw how Dr. Ray was able to take insights from research and apply them to their clinical practice to help extend patients’ lives. This showed me the power of combining research with clinical practice in order to advance patient care.

In many cases, a doctor alone would not be able to improve patient care where there are no existing effective treatments. Likewise, a researcher alone would not have firsthand knowledge of the disease manifestation and would be unable to help implement novel treatments. A combined degree is needed to do either job as effectively as possible and to produce the most meaningful medical advances. This is the type of problem I would like to study in my career, and having a combined MD/PhD degree would give me the best preparation to do so.”

**Supplemental Figure 3.** Female applicant materials

# SAMANTHA [redacted]

University of Maryland, 2100 La Plata Hall, 2121 Farm Drive, College Park, MD 20742

[redacted]@umd.edu


## EDUCATION

**The University of Maryland**, College Park, MD 5/2018

B.S. in Biochemistry, Honors College

GPA 3.72

MCAT 512

**Calvert High School**, Prince Frederick, MD 6/2014

Salutatorian

GPA 3.9

## RESEARCH EXPERIENCE

**Maryland-HHMI Undergraduate Research Fellow** 5/2017-5/2018

Awarded fellowship to conduct a year of research under the direct mentorship of a faculty member, including a stipend of $6000 and up to $1000 in research supplies. Performed biochemical and cell biology experiments to study how signal transduction pathways affect T cell activation in cancer. The goal is to leverage this understanding for novel cancer immunotherapies. This research culminated in my senior thesis.

**Poster presentation** 9/2017

Presented work on senior thesis at CRI-CIMT-EATI-AACR International Cancer Immunotherapy Conference: Translating Science into Survival.

**Summer Internship Program in Biomedical Research** 6/2016-8/2016

Awarded internship to conduct 8-weeks of research at the National Institutes of Health. Identified and characterized genetic mutations in lymphoma and multiple myeloma using genomic and molecular approaches.

**Maryland Student Researcher** 9/2014-12/2014

Worked in a lab researching neurodevelopment in Drosophila for 6 hours per week. Set up crosses and prepared slides for imaging in a genetic screen.

## ACTIVITIES

**America Counts**, Co-president 9/2015-5/2018

Volunteered as a tutor for fourth grade students in local public schools teaching math for 3 hours twice a week. Selected to be co-president during junior year.

**University of Maryland Symphony Orchestra,** first chair cellist 9/2014-5/2018

Played cello in the Symphony Orchestra for all four years of college. Toured and competed with the orchestra internationally. Served as first chair cellist during my senior year.

**Shadowing** 6/2015-8/2015

Shadowed an oncologist for 3 hours per week. Observed patient visits and rounded in the hospital. Shadowed a neurosurgeon for 4 hours per week. Observed surgeries and patient visits.

## AWARDS/HONORS

**Banneker/Key Scholarship** 8/2015-5/2018

Awarded merit scholarship offered to 150 incoming Maryland freshmen that covers full tuition, room and board, and includes admission to the Honors College.

**Dean's List**

Awarded for 6 out of 6 semesters completed for having a GPA of greater than 3.5.

**Excerpt from thesis advisor's letter of recommendation:**
“Samantha began working in my lab a year ago during her junior year and has been an enthusiastic and motivated addition to my team. Although she joined the lab later in her undergraduate career, I have witnessed Samantha impressive ability to quickly learn complex biological concepts and a broad repertoire of laboratory techniques. Her quick mind, collaborative spirit, and rigorous work ethic all demonstrate that she has the potential to become a truly successful physician scientist.”

**Excerpt from why MD/PhD statement:**
“I want to pursue a combined MD/PhD degree because having expertise in both the medical and research fields provides the best opportunity for advancement of the medical field. I shadowed an oncologist who also had a basic research laboratory. I saw how Dr. Ray was able to take insights from research and apply them to their clinical practice to help extend patients’ lives. This showed me the power of combining research with clinical practice in order to advance patient care.

In many cases, a doctor alone would not be able to improve patient care where there are no existing effective treatments. Likewise, a researcher alone would not have firsthand knowledge of the disease manifestation and would be unable to help implement novel treatments. A combined degree is needed to do either job as effectively as possible and to produce the most meaningful medical advances. This is the type of problem I would like to study in my career, and having a combined MD/PhD degree would give me the best preparation to do so.”
